# Delayed coronary obstruction leading to death following balloon dilation in self-expanding aortic valve for acute coronary occlusion: a case report

**DOI:** 10.3389/fcvm.2025.1440231

**Published:** 2025-04-01

**Authors:** Haoran Zhang, Donghui Zhang

**Affiliations:** Cardiovascular Medicine Ward, The Second Affiliated Hospital of Harbin Medical University, Harbin, China

**Keywords:** TAVR, aortic stenosis, coronary obstruction, delay coronary obstruction, acute coronary occlusion, case report

## Abstract

**Background:**

Following transcatheter aortic valve replacement, acute coronary obstruction is infrequent but potentially life-threatening, while delayed coronary obstruction is even more uncommon.

**Case summary:**

A 69-year-old male underwent TAVR and subsequently developed an acute obstruction in the left main coronary artery. Interventional management involved performing percutaneous transluminal coronary angioplasty using balloon dilation on both the left main coronary artery and its ostium. Intravascular ultrasound confirmed successful dilation of the coronary ostium. The patient experienced resolution of symptoms, and ventricular premature beats disappeared on electrocardiogram monitoring. However, the patient unfortunately succumbed to sudden death one month after discharge.

**Conclusion:**

Guidewires for chronic total occlusion may be necessary for patients with severely calcified and stenotic aortic valves that are challenging to navigate. Before undertaking TAVR, precise preoperative evaluation, including accurate risk assessment, multimodal imaging, and thorough planning, is essential. While balloon dilation can provide temporary relief for coronary obstruction, it carries the risk of subsequent delayed coronary occlusion with serious consequences. Chest pain experienced under local anesthesia more directly suggests coronary occlusion.

## 1 Introduction

Coronary artery obstruction (CAO) refers to the occurrence of new or partial/complete obstruction at the coronary ostium during or after transcatheter aortic valve implantation (TAVR), representing a rare but life-threatening complication (1). The incidence of acute coronary obstruction identified by large international multicenter registries is less than 1% (2). Delayed coronary obstruction (DCO) is defined as coronary obstruction occurring after the patient has left the operating room. Jabbour et al. reported an incidence of DCO that is even less common than that for acute obstruction, at approximately 0.22% (3). Furthermore, most DCO events transpire during self-expansion rather than balloon expansion. Fifty percent of these events occur within the first 24 h post-TAVR, and 15% within the first week. A case of acute coronary occlusion following aortic valve implantation, which was successfully treated with balloon angioplasty and subsequently developed delayed obstruction one month later, is reported here.

## 2 Case descriptions

**Figure 1:**
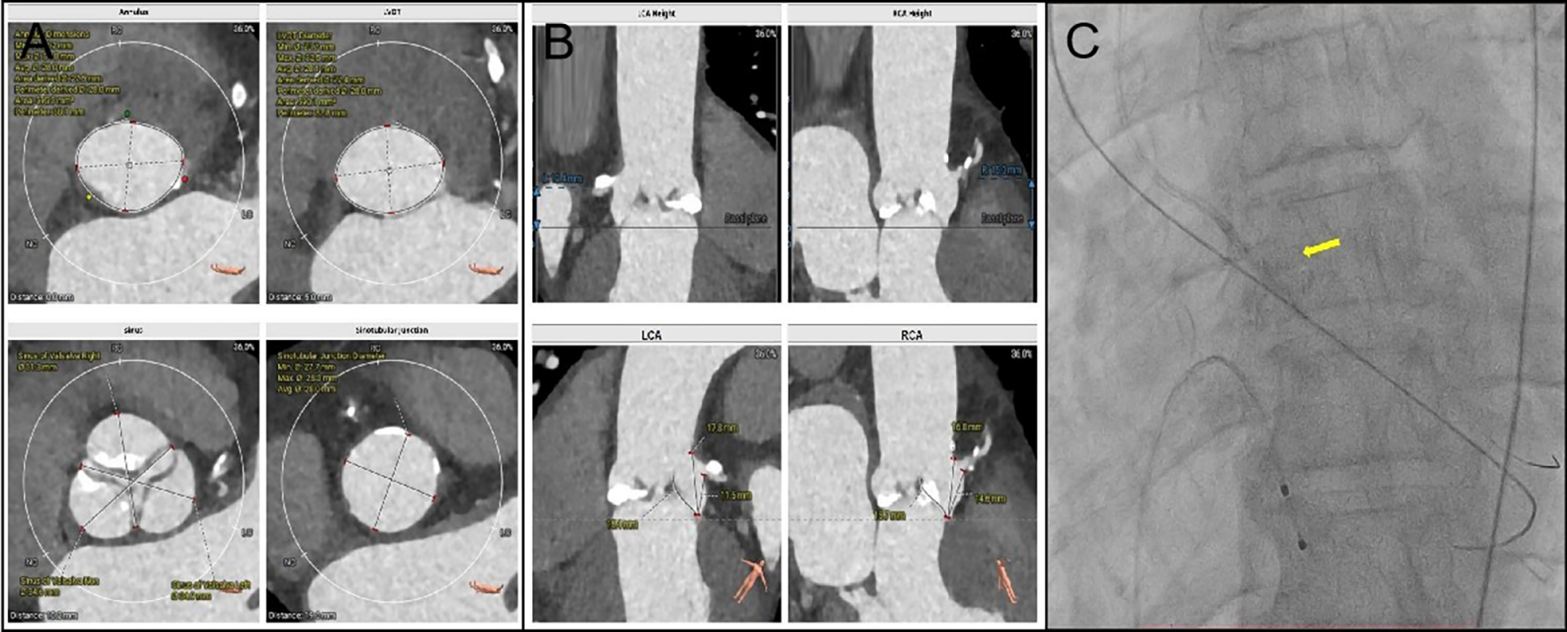
**(A)**: Annulus dimensions Min: 24.2 mm; Max: 31.8 mm; Avg: 28.0 mm; Area derived: 27.5 mm; Perimeter derived: 28.0 mm; Area: 595.3 mm^2^; Perimeter: 88.1 mm. LVOT Diameter Min: 23.7 mm; Max: 32.5 mm; Avg: 28.1 mm; Area derived: 27.4 mm; Perimeter derived: 28.0 mm; Area: 590.1 mm^2^ and Perimeter: 87.8 mm. Sinus of valsalva Left: 34.9 mm; Sinotubular Junction Diameter Min: 27.7 mm; Max: 28.3 mm and Avg: 28.0 mm. **(B)** LCA Heigh 12.4 mm. Left Leaflets Length: 15.4 mm. **(C)** The PILOT 200 wire managed to cross the valve, followed by a 4.0 mm × 15 mm balloon to dilate the aortic valve. The yellow arrow is the balloon.

A 69-year-old male diagnosed with severe aortic valve stenosis and NYHA IV heart failure symptoms was admitted to the Department of Cardiology, Second Affiliated Hospital of Harbin Medical University. The preoperative computed tomography evaluation revealed accessible femoral artery entry points, with the valve annulus area and perimeter recorded at 595.3 mm^2^ and 88.1 mm, respectively, suitable for a 32 mm VenusA Valve implantation (Figures 1A,B). The coronary artery CTA identified proximal stenosis in both the left main stem and the right coronary ostium (Supplementary Figure S1). Following discussions and the patient's refusal of surgical intervention, it was agreed to proceed with a combined single-stage surgery of coronary PCI and transfemoral TAVR (The patient was instructed to take 300 mg of aspirin and 300 mg of clopidogrel prior to the surgery). With the patient's consent, Firebird 2 stents were placed in the right coronary artery and left main stem, and a 32 mm VenusA Valve was implanted.

The procedure was conducted under conscious sedation. Initial the standard straight-tip crossing wire could not traverse the valve, and the 6F AL1 catheter could not be advanced due to wire displacement during repeated efforts. Success was achieved by re-crossing the valve and switching to a 5F AL1 catheter, which allowed entry into the left ventricle. Despite changing to a J-tip wire, the 5F pigtail catheter still failed to progress into the left ventricle. Adopting a radial artery approach, the PILOT 200 wire managed to cross the valve, followed by a 4.0 mm × 15 mm balloon to dilate the aortic valve, which allowed the pigtail catheter to enter the left ventricle successfully (Figure 1C).

**Figure 2:**
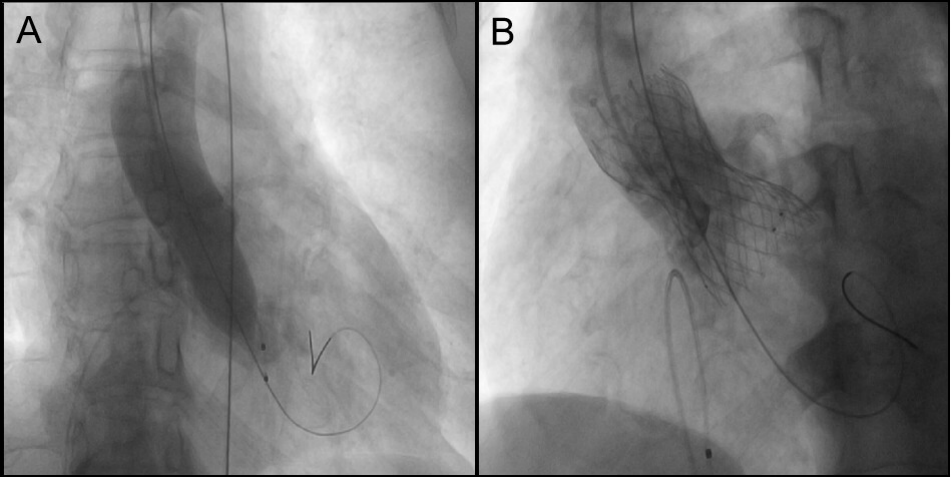
**(A)** 22 mm balloon was employed for aortic valve pre-dilatation. Imaging then demonstrated the relationship between the aortic root and the left coronary artery. **(B)** Imaging indicated a gap between the native valve and the left coronary artery ostium at the aortic sinus base. The yellow curve represents the gap between the leaflets and STJ.

**Figure 3:**
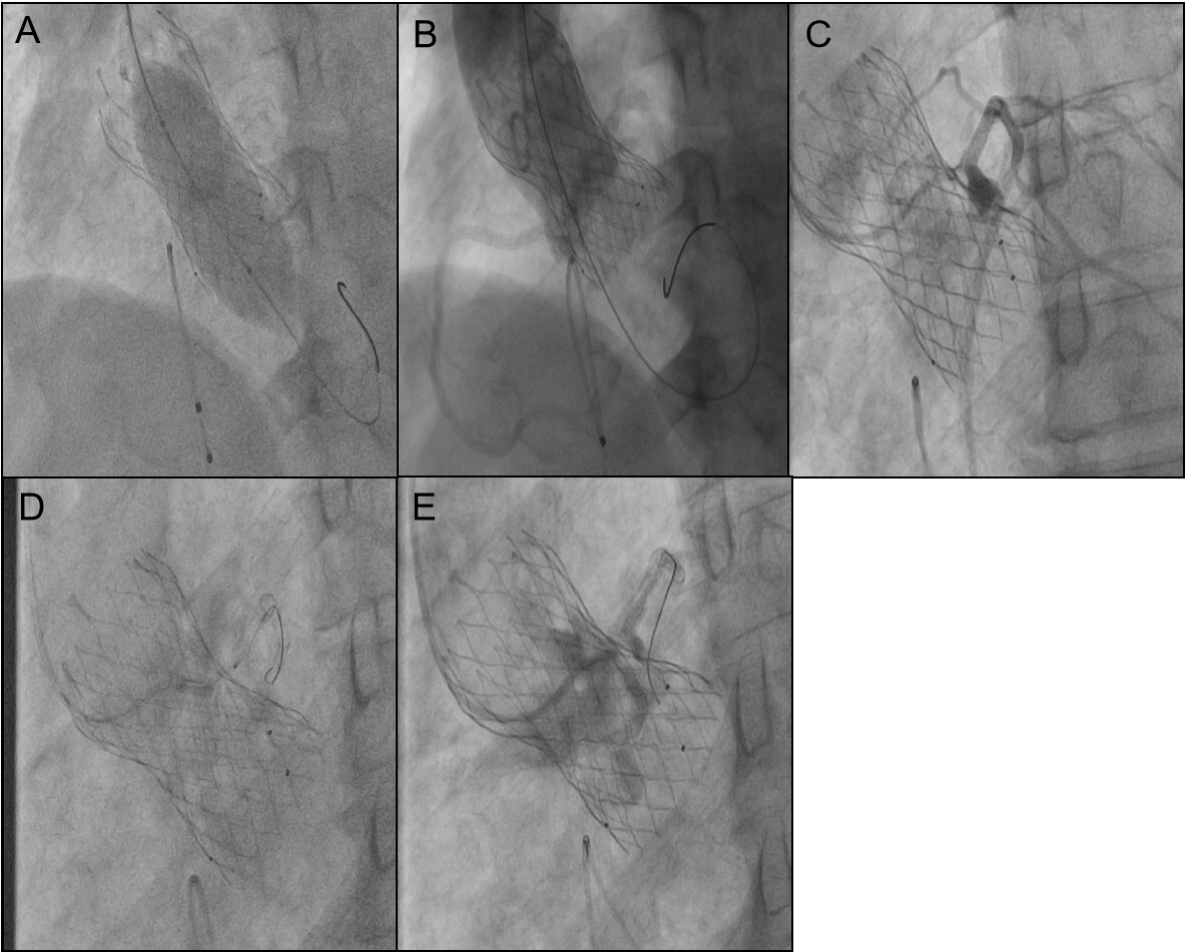
**(A)** 25 mm balloon was used for further dilation due to the inadequate expansion of the valve stent. **(B)** Immediate imaging confirmed blood flow in the left coronary artery. **(C)** Obstruction in the left coronary artery tery. The gap between the leaflets and STJ is tiny. **(D)** Dilation of the left coronary ostium and the obstructed native valve was performed using 2.0 mm, 2.5 mm, and 3.0 mm balloons. **(E)** Angiography shows blood flow passing through.

Implant a 3.5 mm×29 mm stent into the right coronary artery and a 3.5 mm × 24 mm stent into the left main trunk (Supplementary Figure S2). Post-coronary stent implantation, a 22 mm balloon was employed for aortic valve pre-dilatation. Imaging then demonstrated the relationship between the aortic root and the left coronary artery with the pigtail catheter in place (Figure 2A). A 32 mm VenusA Valve was subsequently implanted, and imaging indicated a gap between the native valve and the left coronary artery ostium at the aortic sinus base (Figure 2B). A 25 mm balloon was used for further dilation due to the inadequate expansion of the valve stent, and immediate imaging confirmed blood flow in the left coronary artery (Figures 3A,B). However, three minutes later, the patient exhibited significant chest discomfort and pain, with frequent premature ventricular contractions noted on cardiac monitoring. Rapid imaging detected an obstruction in the left coronary artery (Figure 3C). Efforts were made to navigate a 6F AL1 catheter through the prosthetic valve mesh to access the left coronary artery ostium, and a Pilot 50 wire was extended to the distal left anterior descending artery. Further dilation of the left coronary ostium and the obstructed native valve was performed using 2.0 mm, 2.5 mm, and 3.0 mm balloons (Figures 3D,E). IVUS assessment showed the coronary ostium no stenosis, indicating a marked improvement in coronary blood flow. The patient passed away suddenly one month after the procedure. For details, see Table 1.

## 3 Timeline

**Table 1:** Timeline.

Date	Medical procedures
2023 December	The patient diagnosed with severe aortic valve stenosis and NYHA IV heart failure.
24 h before surgery	Cardiac ultrasound and aortic CTA: evaluating the condition of the patient's valve annulus and coronary artery.
Day of surgery: 8 am	The PILOT 200 wire managed to cross the valve, followed by a 4.0 mm × 15 mm balloon to dilate the aortic valve.
Day of surgery: 8:30 am	Implant a 3.5 mm × 29 mm stent into the right coronary artery and a 3.5 mm × 24 mm stent into the left main trunk.
Day of surgery: 9 am	A 22 mm balloon was employed for aortic valve pre-dilatation; A 32 mm VenusA Valve was subsequently implanted; A 25 mm balloon was used for further dilation.
Day of surgery: 9:03 am	The patient exhibited significant chest discomfort and pain, with frequent premature ventricular contractions noted on cardiac monitoring. Rapid imaging detected an obstruction in the left coronary artery.
Day of surgery: 9:45 am	Further dilation of the left coronary ostium and the obstructed native valve was performed using 2.0 mm, 2.5 mm, and 3.0 mm balloons.
3 days after the surgery	The patient discharged from cardiac center.
1 month after the surgery	The patient passed away.

## 4 Discussion

This case highlights a scenario where there was difficulty navigating a pigtail catheter across the valve, followed by a coronary artery obstruction subsequent to stent deployment. The issue with the pigtail catheter likely arose from severe constriction and calcification of the aortic valve. Initially, a super-slip straight-tip crossing wire could not traverse the valve; however, switching to a CTO wire with a flexible tip and slimmer profile allowed for successful passage through the constricted valve. This adjustment facilitated balloon dilation, enlarging the valve area and enabling the catheter's progression. This provides us with a solution, especially when encountering challenging anatomical features such as valve calcification and stenosis.

Regarding the cause of the patient's death, we suspect it may have been due to delayed coronary obstruction. Acute coronary obstruction generally results from the displacement of native valve leaflets above the coronary ostium due to stent valve compression or direct blockage by the TAVR valve's prosthetic skirt. This can lead to severe complications that are potentially fatal, necessitating quick diagnosis and intervention (4, 5). Thus, measurements of aortic SOV and coronary artery height are critical in assessing the risk of coronary artery obstruction post-TAVR. Research suggests that features like Viv, an SOV smaller than 30 mm, or RCA/LCA shorter than 12 mm pose a high risk for obstruction (1). In this instance, the patient's left coronary artery height measured at 12.4 mm and SOV at 34.9 mm, not aligning with the high-risk anatomical criteria. Recent proposals for assessing the risk of post-TAVR coronary artery obstruction include novel metrics combining VTC, VTSTJ, and the difference in height between the STJ and the leaflet length. Patients with THV-coronary distances under 3.0 mm, THV-to-STJ distances under 1.0 mm, or a negative leaflet-STJ mismatch are at an elevated risk of complete TCO (53% incidence) (6). For this patient, the preoperative assessment indicated a VTC of 5.6 mm, a VTSTJ of 1.2 mm, and a leaflet-STJ mismatch of 2.4 mm, suggesting a low-risk profile (Supplementary Figure S3).

Coronary obstruction, in this case, occurred after balloon expansion, potentially due to the compression and subsequent distortion or tearing of native leaflets, which diminished the gap between the native leaflets and the STJ. Although initial imaging post-valve deployment showed a clear distance between the native valve and the coronary ostium with some blood flow through the left coronary artery, within 3 min, the patient experienced significant chest pain and frequent premature ventricular contractions. Given that local anesthesia was used and the patient remained conscious, the sudden onset of severe chest pain was a clear indicator of coronary obstruction. Angiography performed with an AL1 catheter showed the native leaflets nearly adhering to the STJ, impairing blood flow essential for myocardial perfusion and precipitating the chest pain. The coronary artery, while not fully occluded, did not result in severe outcomes like ventricular fibrillation. A CTO wire was reintroduced through the narrowed gap, and smaller balloons were sequentially employed to reinstate blood flow, which was confirmed by an intravascular ultrasound as having an adequate opening area. The decision for balloon-only dilation to manage the blocked coronary ostium was driven by several factors: the urgency required a method easily tracked by the imaging catheter's mesh to engage the left coronary artery ostium, particularly as the coronary stent could not navigate past the imaging catheter. Additionally, since the infarction emerged post-prosthetic valve dilation, angiography did not initially show complete coronary obstruction. Re-dilation of the space between the native leaflets and the STJ likely returned it to its state following stent placement. Following the dilation, IVUS assessments showed a satisfactory opening area, and the patient's symptoms eased. Another reason, there is a stent in the left main trunk itself. If a window stent is chosen, there is also a risk of thrombosis in the left main trunk multi-layer stent.

However, it was discovered that the patient had died suddenly one month after surgery. The suspected causes of death include delayed coronary occlusion or the retraction of the original leaflets following balloon expansion, which may have led to the re-occlusion of the coronary ostium. Other possible causes are compression by aortic root hematoma, endothelialization of the prosthetic valve, thrombosis of the leaflets, or inflammatory proliferation in the aortic root. These are all TAVR-related causes of death. However, considering that the patient also underwent coronary stent implantation, stent-related causes of death cannot be excluded. Further discussions among the surgical team indicated that the use of fenestrated stents might have prevented such severe outcomes. Moreover, it may be necessary to switch from local to general anesthesia with intubation followed by transesophageal echocardiography to evaluate the risk of coronary obstruction. Research indicates that patients with LCA ostial obstruction exhibit higher peak diastolic flow velocities in the LM post-TAVR and a greater ratio of flow velocities before and after TAVR compared to those without such obstruction (7). These parameters are lower in hemodynamically unstable patients with LCA obstruction than in stable ones. Additionally, IVUS results in assessing coronary obstruction may not be sufficiently accurate. Certain techniques can intentionally induce the rupture of small leaflets located near the coronary artery ostium to prevent coronary artery obstruction. The BASILICA (Bioprosthetic or Native Aortic Leaflet Intentional Laceration to Prevent Coronary Artery Occlusion) technique was first described in 2018 and has achieved a notable success rate (8).

The discussion above primarily focuses on the causes of death associated with TAVR (transcatheter aortic valve replacement). However, given that the patient also underwent coronary artery stent implantation, the possibility of stent-related mortality cannot be excluded. After discharge, the patient has been regularly taking aspirin 100 mg Qd and clopidogrel 75 mg Qd, along with heart failure medications including spironolactone 20 mg Qd, sacubitril/valsartan 50 mg Bid, and metoprolol 47.5 mg Qd. Although regular use of antiplatelet drugs should reduce the risk of stent thrombosis, clopidogrel resistance remains a potential concern, and platelet inhibition testing was not conducted to rule out this possibility (9). Additionally, the occurrence of bleeding events, such as sudden intracranial hemorrhage, cannot be completely excluded. Evidence suggests that routine PCI prior to TAVR does not confer a significant benefit in terms of short- or mid-term mortality and may actually increase the risk of bleeding events (10). Moreover, heart failure patients are at increased risk for malignant arrhythmias, although the 24 h Holter monitoring upon admission did not show any abnormal findings such as prolonged QT, premature ventricular contractions, or bundle branch blocks.

In summary, the lessons learned are as follows: CTO wires are suitable for patients with severe calcification and narrowing of the aortic valve. Preoperative assessment, including accurate risk evaluation, precise multimodal imaging, and meticulous planning, is essential prior to TAVR interventions. Balloon dilation can temporarily alleviate coronary obstruction; however, ongoing expansion of the metallic stent and retraction of native leaflets, coupled with thrombotic factors, may result in severe outcomes such as delayed coronary occlusion. Currently, most centers favor general anesthesia for TAVR procedures, as patients under conscious sedation may exhibit symptoms of chest pain more directly linked to coronary obstruction compared to traditional indicators of coronary obstruction, which include persistent severe hypotension, electrocardiographic alterations (acute myocardial infarction often shows no ST-segment changes), and ventricular arrhythmias. Consequently, considering complication management, local anesthesia sedation might be a more appropriate anesthetic approach.

## Data Availability

The original contributions presented in the study are included in the article/Supplementary Material, further inquiries can be directed to the corresponding author.
