# Supplementary material for: Delayed coronary obstruction leading to death following balloon dilation in self-expanding aortic valve for acute coronary occlusion: a case report
Source: Front Cardiovasc Med. 2025 Apr 1;12:1440231. doi: 10.3389/fcvm.2025.1440231 (PMC11996906; doi:10.3389/fcvm.2025.1440231)
Supplement: Supplementary file 1 [file Datasheet1.pdf]

Supplementary Material

1 Supplementary Figures

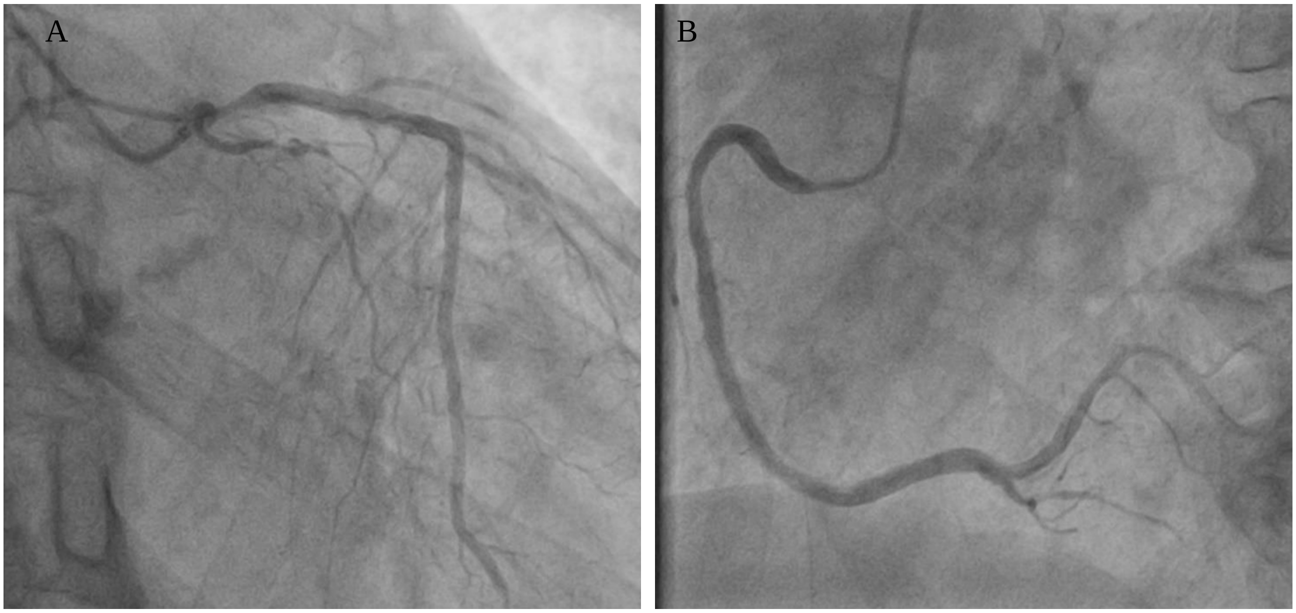

Supplementary Figure 1. A: Proximal stenosis in the left main stem. B: Proximal stenosis in the right coronary.

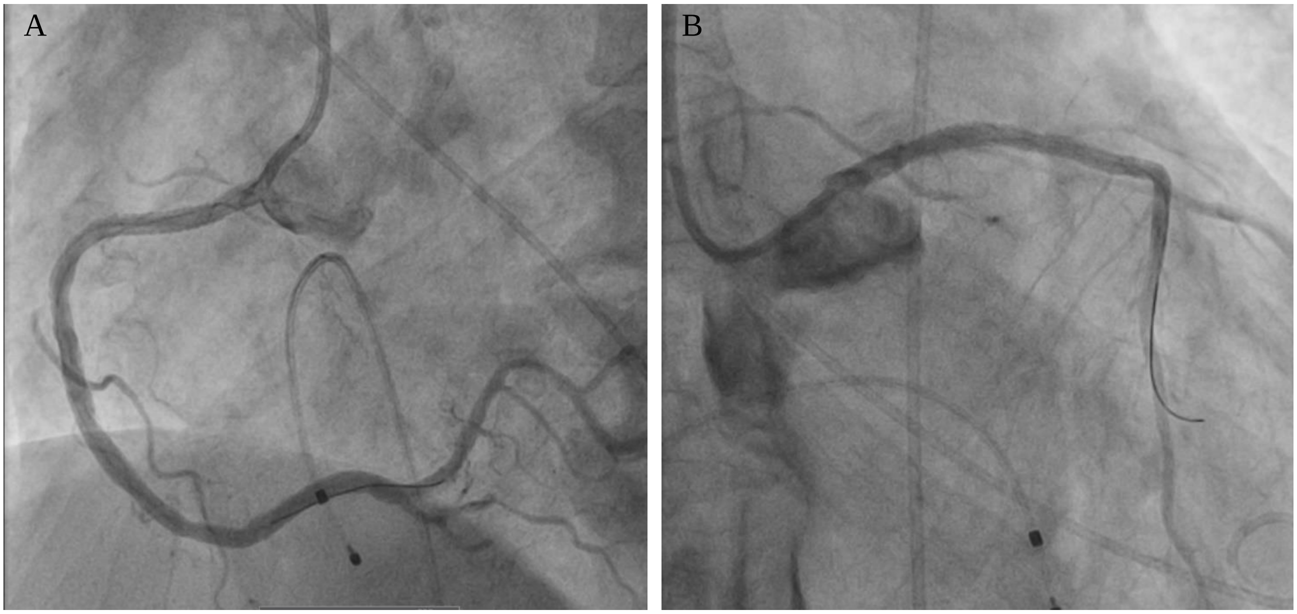

Supplementary Figure 2. A: Implant a 3.5 mm x 29 mm stent into the right coronary artery. B: A 3.5 mm x 24 mm stent into the left main trunk.

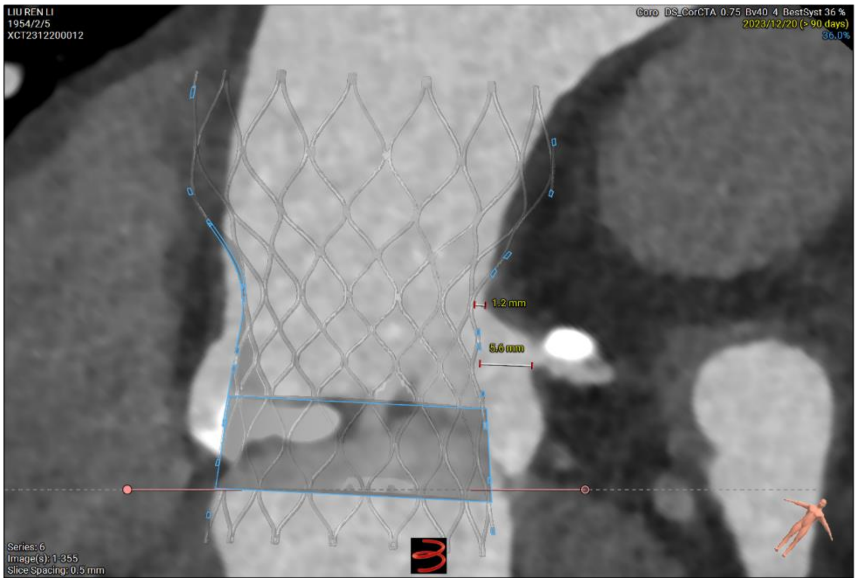

Supplementary Figure 3. VTC 5.6 mm, VTSTJ 1.2 mm, and the leaflet-STJ mismatch of 2.4 mm.
